# A Dosimetric Comparison of Primary Chemoradiation Versus Postoperative Radiation for Locally Advanced Oropharyngeal Cancer

**DOI:** 10.7759/cureus.1858

**Published:** 2017-11-17

**Authors:** Stanley K Woo, Chad Freeman, Brock J Debenham

**Affiliations:** 1 Department of Oncology, University of Alberta

**Keywords:** oropharyngeal cancer, radiation, surgery, head and neck cancer, head and neck cancer, hpv

## Abstract

Introduction

Advanced-stage oropharyngeal cancer can be treated with primary chemoradiation (CRT) or primary surgery with adjuvant radiotherapy, both with similar survival outcomes. Though primary CRT prescribes a higher dose, adjuvant radiation requires irradiating the surgical bed, which may increase the high dose planned target volume (PTV). We hypothesize that the integral dose to the neck and dose to critical structures will be lower with primary CRT than adjuvant radiotherapy.

Methods

We selected the last 18 patients who underwent surgery and adjuvant radiotherapy at one institution between July 2015 and August 2016 with American Joint Committee on Cancer (AJCC) stage III or IVA oropharyngeal squamous cell cancer. Primary CRT treatment plans were created on the patients’ preoperative computed tomography (CT) scans and prescribed 70 Gy in 33 fractions, while postoperative plans were prescribed 60 Gy in 30 fractions. The radiation doses received by organs at risk for each primary CRT plan were compared to the corresponding adjuvant radiation plan.

Results

Primary CRT plans had significantly smaller high dose PTV than adjuvant radiation plans (187.3 cc (95% CI 134.9-239.7) and 466.3 cc (95% CI 356.7-575.9), p<0.0001). The neck integral dose was lower in 14 of 18 plans using primary CRT, although this was not statistically significant (p=0.5375). The primary CRT plans had lower mean doses to ipsilateral (31.8 Gy (95% CI 27.5-36.0) vs 39.3 Gy (95% CI 35.4-43.1), p=0.0009)) and contralateral parotid glands (22.5 Gy (95% CI 22.1-22.8) vs 27.6 Gy (95% CI 23.4-31.8), p=0.0238) and larynx (20.7 Gy (95% CI 19.3-22.2) vs 40.2 Gy (95% CI 30.8-46.6), p<0.0001).

Conclusion

Primary CRT offered a decreased neck integral dose, though it was statistically insignificant. Primary CRT plans reduce mean dose to larynx and parotid glands in comparison to postoperative radiation, which may result in lower toxicities. Clinical trials comparing primary CRT and primary surgery are warranted to compare patient toxicities.

## Introduction

Approximately 70% of oropharyngeal cancer patients present at advanced stages [1]. For these patients, two common treatment modalities exist: primary surgery with postoperative radiation (with or without chemotherapy) and primary chemoradiation (with or without salvage surgery). Both modalities achieve comparable survival outcomes for advanced stage oropharyngeal patients [2], but there is still no consensus on a preferred treatment modality.

Modalities are selected based on anatomic location, patient factors/values, and physician influences. Despite similar tumour control rates, surgery causes significantly more complications that require remedial surgery, such as fistulas or permanent gastrostomies [3]. Boscolo-Rizzo et al. [4] found that chemoradiation had significantly higher long-term quality of life scores than surgery with postoperative radiation. A retrospective single centre study found that surgery followed by chemoradiation gave the patient population the best survival rates compared to surgery with postoperative radiotherapy or chemoradiotherapy alone [5]; however, combining more treatment modalities often increases patient morbidity. Therefore, a trade off exists between tumour control and reduced side effects that physicians and patients must consider before selecting a treatment.

Before selecting a preferred modality, patients must consider the radiation dose to organs at risk (OAR) in the head and neck, such as the parotid glands, larynx, and mandible. While primary chemoradiation (CRT) prescribes a higher dose, adjuvant radiation (RT) may deliver more radiation to OAR because the entire post-surgical bed requires irradiation. Irradiating larger tissue volumes can increase the number and severity of side effects.

We hypothesize that the integral dose to the neck and dose to critical structures will be lower with primary CRT than adjuvant radiotherapy. Evaluating the difference in mean dose and maximum dose to OAR and the total integral dose between CRT and adjuvant RT will provide additional insight into the optimal treatment modality for locally advanced oropharyngeal cancer patients.

## Materials and methods

The study was submitted for a Research Ethics Board (REB) review and follows the Tri-Council Policy Statement: Ethical Conduct for Research Involving Human Subjects (TCPS2), as data collection involved retrospective patient information such as computed tomography (CT) scans and radiotherapy treatment plans from the Cross Cancer Institute (Edmonton, Canada). The REB determined that ethics approval was not required as this is a quality improvement based study.

**Figure 1:**
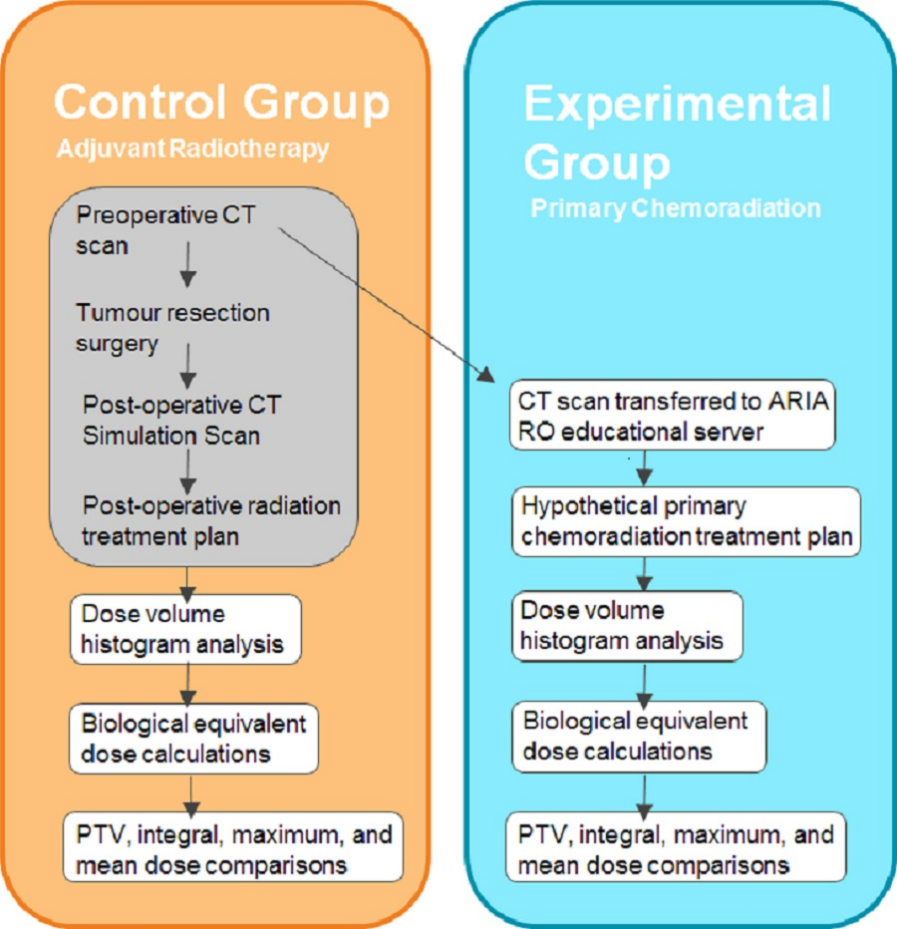
Experimental Design Description of our methodology and how we defined our control and experimental group. The control were the actual adjuvant RT plans, whereas the experimental group were the primary CRT plans. The grey shaded rectangle represents steps that were completed prior to this study. (CT = computed tomography, PTV = planning tumor volume, RO = radiation oncology).

Using a retrospective cohort, we compared the total integral dose and dose to OAR that patients received when they were treated with adjuvant RT to a theoretical CRT plan using their preoperative CT scans. This study included both an experimental and control group as summarized in Figure 1.

The last 20 consecutive patients with American Joint Committee on Cancer (AJCC) stage III or IVA oropharyngeal cancer who underwent primary surgery and postoperative radiotherapy for their disease at our centre between July 2015 and August 2016 were included in our study. Apart from the tumour resection surgery, patients had no previous head and neck surgeries for malignancies and no previous head and neck radiotherapy treatments.

We obtained the postoperative radiotherapy plans (60 Gy in 30 fractions delivered daily) and dose-volume-histograms (DVH) from the patients noted above to serve as our control group. Our standard procedure is to treat the post-surgical bed and involved lymph node levels to 60 Gy, and the uninvolved neck to 54 Gy. The radiation treatments were planned and completed at the Cross Cancer Institute. Each patient’s plans and images were anonymized and assigned a random identification number.

For our experimental group, the patients’ preoperative diagnostic CT scans were transferred to the ARIA® oncology information system for radiation oncology, which contains the radiotherapy treatment planning software used clinically at the Cross Cancer Institute. The parotid glands, submandibular glands, mandible, esophagus, larynx, and pharyngeal constrictor muscles were contoured as critical structures using the contouring guidelines from the Radiation Therapy Oncology Group (RTOG) protocol 1016. The RTOG 1016 protocol contains planning guidelines and objectives for target volumes and critical structures for primary chemoradiation of advanced stage oropharyngeal patients. A treatment couch structure was added to each plan to simulate the radiotherapy units. We generated volumetric arc therapy plans, which was the same technique used in postoperative plans, with the Eclipse 3D planning system (version 13.6, Varian Medical Systems, Palo Alto, CA). The dose prescription was different, 70 Gy in 33 fractions delivered daily, but the dose algorithm and plan normalization (95% of PTV received 100% of the dose) were maintained with the adjuvant RT plans to provide intrapatient consistency. Normal tissue optimizations were the same between postoperative and preoperative planning to reduce bias. Clinical target volume (CTV) to PTV margins were 0.5 cm in both groups. To account for the differences in fractionation, integral doses and OAR doses were converted to equivalent doses in 2 Gy fractions (EQD2).

As a form of quality assurance, a head and neck radiation oncologist and dosimetrist from the Cross Cancer Institute reviewed each contour and plan. The constructed primary CRT plan was compared to the patient’s postoperative plan to assess the difference in integral dose and dose to OARs. Integral dose to the overall neck was calculated by multiplying the volume between the cochleas and the cricoid cartilage with the mean dose of that volume.

Data were analyzed using paired T-tests to determine any significant differences between the two regimes for each patient. A p-value of < 0.05 was taken to be statistically significant. To determine our sample size, it was assumed that there would be a difference in integral dose between the two groups of 15% with a standard deviation of 10 for each group. Assuming a type I error of 0.05 and a type II error of 0.20, we required a total of 20 patients for statistical significance.

## Results

Data were collected for 20 patients, but two patients were omitted from analysis because of positional and scan size issues. Therefore, 18 patients were analyzed. Table 1 lists the patient characteristics of our sample group.

**Table 1:** Patient Demographics, Pathology, and Surgery Results

Demographic	Characteristics
Age	Median 64 (range 46-76)
Sex	94.7% male
Subsite	50% base of tongue 27.8% tonsil 22.2% base of tongue and tonsil
Clinical AJCC stage	5.3% III 94.7% IVA
p16 status	83.3% positive 16.7% negative
Extracapsular extension (ECE)	66.7% positive 33.3% negative
Lymphovascular invasion (LVI)	38.9% positive 61.1% negative
Perineural invasion (PNI)	33.3% positive 66.7% negative
Positive margins	22.2% positive 77.8% negative
Percutaneous endoscopic gastrostomy tube inserted	44.4% after surgery (before RT) 11.1% during RT

The average high dose PTV volumes for the plans made on the patients’ preoperative CT scans were 40.2% smaller compared to the postoperative plans, as seen in Table 2.

**Table 2:** Comparison of High Dose PTV Volumes and Integral Dose to the Head and Neck Region Between the Primary RT Cohort and Adjuvant RT Cohort *Note: *All doses converted to 2 Gy fraction equivalent. P-value </= 0.05 indicates statistical significance.

	Primary RT	Adjuvant RT	P value
High dose PTV volume (cc)	187.3 (95% CI 134.9-239.7)	466.3 (95% CI 356.7-575.9)	p < 0.0001
Integral dose to the neck (Gy*L)	152.6 (95% CI 130.3-174.9)	156.6 (95% CI 134.7-178.5)	p=0.5375

The DVH comparison of the control group versus experimental plans revealed significant differences. Table 3 contains the mean and maximum dose averages for the critical structures analyzed between the two treatment groups. The maximum doses for the ipsilateral parotid gland, the mandible, the pharyngeal constrictor muscles, and the spinal cord were significantly lower for the adjuvant RT group. Also, the mean dose to the spinal cord was also significantly lower for the adjuvant RT group. On average, the primary CRT group had lower mean doses for the ipsilateral and contralateral parotid glands, esophagus, larynx, and mandible, but only doses to the ipsilateral and contralateral parotid glands and larynx were significantly lower than the adjuvant RT plans. The primary CRT group also had lower maximum doses for the contralateral parotid and oesophagus, but these differences were not statistically significant. The difference in the mean pharyngeal constrictor dose and max larynx dose was only 0.4 cGy and 1.1 cGy, respectively. Submandibular glands were resected for most patients so their dose comparisons were omitted. For individual patient doses, refer the Appendix.

**Table 3:** Comparison of Mean and Maximum Doses to Critical Structures Between the Primary RT and Adjuvant RT Cohort *Note: *All doses converted to 2 Gy per fraction equivalent. P-value </= 0.05 indicates statistical significance.

Structure	Primary CRT (Gy)	Adjuvant RT (Gy)	P value
Maximum dose to ipsilateral parotid	76.8 (95% CI 74.8-78.7)	65.7 (95% CI 64.4 to 67.0)	p < 0.0001
Mean dose to ipsilateral parotid	31.8 (95% CI 27.5-36.0)	39.3 (95% CI 35.4-43.1)	p = 0.0009
Maximum dose to contralateral parotid	56.2 (95% CI 52.3-59.8)	58.4 (95% CI 52.6-64.1)	p = 0.4566
Mean dose to contralateral parotid	22.5 (95% CI 22.1-22.8)	27.6 (95% CI 23.4-31.8)	p = 0.0238
Maximum dose to esophagus	46.4 (95% CI 42.3-50.6)	50.8 (95% CI 46.4-55.2)	p = 0.1266
Mean dose to esophagus	24.1 (95% CI 21.9 - 26.3)	29.7 (95% CI 23.3-36.1)	p = 0.0547
Maximum dose to larynx	59.5 (95% CI 53.2-65.8)	60.6 (95% CI 58.8-62.5)	p = 0.7307
Mean dose to larynx	20.7 (95% CI 19.3-22.2)	40.2 (95% CI 30.8-46.6)	p < 0.0001
Maximum dose to mandible	75.7 (95% CI 72.9-78.6)	65.3 (95% CI 64.9-65.7)	p < 0.0001
Mean dose to mandible	37.8 (95% CI 35.3-40.3)	40.6 (95% CI 38.0-43.2)	p = 0.1010
Maximum dose to pharyngeal constrictors	77.4 (95% CI 76.4-78.3)	64.7 (95% CI 64.0 - 65.4)	p < 0.0001
Mean dose to pharyngeal constrictors	56.4 (95% CI 52.9-59.8)	56.8 (95% CI 55.0-58.7)	p = 0.7745
Maximum dose to spinal cord	43.0 (95% CI 42.3-43.7)	40.9 (95% CI 40.1-41.6)	p < 0.0001
Mean dose to spinal cord	31.6 (95% CI 30.4-32.9)	21.1 (95% CI 19.2-22.9)	p < 0.0001

## Discussion

Radiation therapy is commonly used to treat advanced stage oropharyngeal cancer, whether it be used adjuvant to surgery or as the primary modality along with chemotherapy [2]. Beyond survival rates, there are very few studies that compare these two regimes. There is lack of randomized trials investigating quality of life following treatment with chemoradiation or surgery with postoperative radiation; therefore, a preferential treatment option still does not exist for these patients [2-4]. Though Tillman et al. [6] studied a different tumour site with a different method, their results are consistent with ours in that their postoperative RT cohort had a larger PTV, and OARs such as the heart and lungs received a higher dose. Our study provides a similar dosimetric comparison and analysis that suggests that additional controlled studies are needed to further inform the patient’s decision between these two treatment methods. As predicted, our study showed a significantly smaller mean high dose PTV for the primary CRT cohort, which was hypothesized to result in a lower integral dose. The primary CRT plans on average had lower integral doses. In fact, 14 out of 18 primary CRT plans had lower integral doses than their corresponding postoperative plans, although this was not significantly different, possibly due to the small number of patients in this study. Conversely, the results suggest that adjuvant RT is not advantageous over primary CRT in regards to delivering lower integral doses to a patient’s normal tissues.

The mean ipsilateral and contralateral parotid gland dose was reduced by 19% and 18.5% in the primary CRT cohort, respectively. This reduction in dose to the parotids has major implications for the quality life of these patients, as the risk of xerostomia decreases. For every 1 Gy increase in parotid mean dose, salivary function decreases by 5% [7]. If at least one parotid gland receives a mean dose of less than 25.8 Gy, the risk of grade 4 xerostomia is significantly lower. As the primary CRT contralateral parotid gland received less than 25.8 Gy and both adjuvant RT parotid glands received more than 25.8 Gy, we expect the primary CRT cohort to have a significantly lower risk of severe xerostomia, and, therefore, a better quality of life over the long-term.

While most submandibular RTOG 1016 dose targets were achieved for the primary CRT plans, many postoperative RT patients had their submandibular glands removed so dose statistics between the cohorts could not be compared. Though primary CRT delivers radiation to the submandibular glands, the risk of xerostomia due to submandibular irradiation is better than xerostomia from the absence of submandibular glands.

The primary CRT larynx structure had a 48.5% lower mean dose than adjuvant RT. Caudell et al. [8] found that higher mean doses were significantly associated with severe dysphagia at 12 months post-treatment. Patients began to experience aspiration at a mean dose of 41 Gy to the larynx. At doses higher than the threshold, the risk of severe dysphagia is significantly correlated with increasing dosage. With the average mean dose of 40.2 Gy for adjuvant RT and 20.7 Gy for primary CRT, we would expect primary CRT patients to have a lower risk of aspiration. Also, as Caudell et al. [8] only studied primary CRT, postoperative RT patients may experience more severe comorbidities at a mean dose of 41 Gy as patients irradiated postoperatively suffer lower quality of life and more severe pain with the same dose prescription comparison as our study [4].

Our study’s findings show that opting for surgery would not spare advanced oropharyngeal patients of the integral dose and that primary CRT lowers the mean dose to some OARs. Our results also indicated that treating a smaller volume to a higher dose in the primary CRT setting would not increase the risk of developing radiation-related side effects as there is a predicted lower risk of xerostomia and aspiration. In addition to potentially improving quality of life, lower toxicities can decrease appointment and treatment cancellations, which improves outcomes and decreases healthcare costs.

Due to a higher prescribed dose for the primary CRT cohort, we expected higher maximum doses received by many OARs than in the adjuvant RT cohort. The high dose PTV may overlap with some OARs, so maximum doses in those organs are difficult to avoid. The significantly higher maximum dose observed in the primary CRT cohort for the mandible would result in a higher risk of osteoradionecrosis. According to Emami [9], this risk increases above 5% with a point dose greater than 70 Gy. Thankfully, most of the toxicity in head and neck critical structures are based on mean dose rather than point doses.

There were multiple limitations in our study. The primary CRT cohort was planned on diagnostic CT scans, and therefore the patients were not in a traditional RT position with an immobilizing shell with shoulders depressed and chin extended. This may have resulted in dosimetric differences between the cohorts, with a likely negative effect on the primary CRT group’s OAR optimization abilities because these scans had compressed anatomy due to the lack of neck extension position in diagnostic scans. Secondly, although treating the post-surgical bed and bilateral neck for locally advanced oropharyngeal patients who have undergone surgery is standard at our center, it may not be so at other centers. There is evidence that postoperative radiotherapy to the ipsilateral neck may be all that’s needed for patients with N2a-b disease [10].

## Conclusions

In conclusion, primary CRT offered a lower total integral dose to the neck on average, although this was not statistically significant. Given that primary CRT plans prescribed a higher dose, higher maximum organ doses were expected. However, lower mean organ doses suggested that primary CRT plans spare more larynx and parotid gland than postoperative radiation, which may result in lower overall toxicity to the patient. Randomized clinical trials are necessary to further validate these findings and better inform the management decisions of advanced stage oropharyngeal cancer patients.

## Appendices

Raw Data and Non-2 Gy Fraction Equivalent Doses for Each Patient and Critical Structure

**Table 4:** Mean and Maximum Doses to Each Patient’s Ipsilateral and Contralateral Parotids for Primary Radiotherapy (RT) and Postoperative Radiotherapy (PO).

Patient	Max Ipsilateral Parotid (Gy)		Mean Ipsilateral Parotid (Gy)		Max Contralateral Parotid (Gy)		Mean Contralateral Parotid (Gy)	
	RT	PO	RT	PO	RT	PO	RT	PO
1	75.978	63.095	38.563	54.682	54.718	57.448	25.341	37.933
2	74.210	64.622	36.730	38.668	53.402	65.050	25.344	37.309
3	75.845	67.223	36.689	45.726	53.977	64.805	25.258	39.933
4	73.953	62.092	30.267	37.896	61.910	60.589	24.906	30.711
6	76.897	68.343	25.758	40.633	70.347	64.245	25.124	37.533
7	73.834	59.448	39.063	37.998	56.220	54.438	25.947	19.936
8	75.258	64.982	51.725	50.103	54.314	60.896	25.272	26.159
9	69.377	64.032	25.509	34.649	51.962	60.288	24.414	26.411
10	75.626	66.059	40.951	32.408	54.544	55.916	25.277	24.255
11	79.245	65.102	34.893	36.877	55.340	60.265	24.829	37.243
12	71.596	63.947	29.201	39.117	55.832	58.189	25.430	30.013
13	67.381	64.863	25.424	42.677	53.720	60.184	24.217	29.400
14	75.950	64.450	23.626	41.196	76.040	64.450	23.286	41.196
15	75.710	64.135	43.028	38.344	53.619	62.386	24.418	30.836
17	81.209	65.170	48.659	59.380	56.979	62.166	25.123	19.327
18	74.371	63.987	24.644	39.116	58.963	63.987	24.644	26.565
19	75.496	66.841	35.684	40.245	55.770	16.705	26.419	7.918
20	78.995	68.918	27.872	33.896	54.183	58.292	25.656	35.531

**Table 5:** Mean and Maximum Doses to Each Patient’s Mandible and Pharyngeal Constrictors (PC) for Primary Radiotherapy (RT) and Postoperative Radiotherapy (PO)

Patient	Max Mandible (Gy)		Mean Mandible (Gy)		Max PC (Gy)		Mean PC (Gy)	
	RT	PO	RT	PO	RT	PO	RT	PO
1	68.856	65.511	31.564	44.637	75.970	64.975	55.198	60.188
2	73.175	64.353	37.749	46.025	76.622	65.203	55.609	60.253
3	75.541	64.873	38.780	39.858	74.602	65.756	55.975	60.891
4	74.458	63.852	42.278	37.272	74.226	63.321	64.132	59.135
6	77.206	63.510	46.251	46.686	76.499	64.414	66.692	61.856
7	75.732	63.338	42.309	43.687	74.998	63.101	60.134	59.703
8	75.758	65.564	44.287	35.177	76.066	64.929	60.220	61.241
9	76.277	63.686	47.105	40.354	72.836	63.250	54.780	52.783
10	75.696	65.306	39.936	47.274	75.666	64.125	54.709	53.728
11	77.837	64.064	40.812	49.547	74.624	65.323	56.869	57.235
12	63.747	63.776	33.739	42.059	75.202	64.009	56.302	59.157
13	60.700	64.145	32.319	45.653	76.053	63.792	55.648	60.482
14	76.805	63.892	45.635	47.381	74.715	64.14	60.078	61.575
15	75.589	64.646	40.760	45.521	75.323	63.709	59.134	60.138
17	79.150	65.395	44.039	46.895	76.996	63.544	56.490	55.535
18	76.710	64.474	45.603	41.087	80.179	60.450	57.841	48.406
19	76.005	65.394	37.279	31.915	73.515	64.288	55.708	37.185
20	75.644	65.120	35.364	36.171	75.771	63.771	58.005	52.512

**Table 6:** Mean and Maximum Doses to Each Patient’s Larynx and Esophagus for Primary Radiotherapy (RT) and Postoperative Radiotherapy (PO)

Patient	Max Larynx (Gy)		Mean Larynx (Gy)		Max Esophagus (Gy)		Mean Esophagus (Gy)	
	RT	PO	RT	PO	RT	PO	RT	PO
1	55.211	57.797	25.828	46.85	48.947	58.157	29.360	45.744
2	53.781	62.123	25.256	45.202	41.628	52.123	30.268	32.025
3	53.460	60.269	25.091	24.870	58.375	34.771	22.414	16.311
4	63.436	58.689	25.963	52.500	42.979	56.451	30.257	49.662
6	68.064	64.414	25.584	61.856	59.767	62.881	29.832	51.394
7	51.546	61.068	24.969	35.542	48.679	43.772	29.852	24.903
8	77.158	56.392	24.675	30.797	57.147	51.000	17.937	13.933
9	53.470	56.194	24.474	28.057	44.746	52.477	28.725	13.721
10	49.455	60.581	24.688	34.448	47.729	50.196	29.819	25.512
11	67.345	66.255	20.277	45.123	61.083	62.817	24.290	38.646
12	62.682	63.936	20.934	58.526	50.893	59.882	25.541	28.115
13	49.980	63.268	19.560	55.714	52.023	57.241	28.577	55.714
14	55.298	63.130	18.920	58.253	44.076	58.117	26.263	34.192
15	51.699	63.292	19.633	34.621	46.290	47.428	28.781	32.260
17	73.034	60.214	27.990	31.747	49.850	46.782	29.616	29.198
18	87.904	57.702	26.171	42.294	48.628	45.403	29.392	35.469
19	57.484	55.129	18.715	25.358	40.465	37.482	26.139	21.093
20	50.654	58.446	19.079	42.728	28.671	55.548	13.233	24.896

**Table 7:** Mean and Maximum Doses to Each Patient’s Spinal Cord, Integral Dose (ID), and PTV size for Primary Radiotherapy (RT) and Postoperative Radiotherapy (PO)

Patient	Max Cord (Gy)		Mean Cord (Gy)		ID (Gy/L)		PTV (cc)	
	RT	PO	RT	PO	RT	PO	RT	PO
1	46.862	43.608	37.008	27.592	96.20714	108.7386	61.7	442.3
2	44.382	42.434	31.831	22.836	114.82917	133.9186	166.2	448.9
3	44.891	43.280	34.376	20.895	115.65425	120.4623	174.7	390.5
4	44.243	42.610	29.730	21.671	129.98775	135.6595	117.0	121.2
6	46.612	42.823	37.577	26.060	143.41090	150.5132	315.5	981.7
7	43.399	40.033	34.694	18.327	120.03611	92.2390	146.0	272.7
8	46.314	44.487	29.391	27.487	129.75098	129.1895	267.9	496.2
9	44.649	39.295	36.507	21.422	137.63596	135.1606	157	297.8
10	43.798	43.371	33.189	21.675	97.36787	101.2248	139.4	443.8
11	45.685	43.002	31.444	26.922	77.82079	85.48116	110.3	774.6
12	46.341	42.588	34.935	26.45	117.54916	123.2601	171.2	378.3
13	46.765	44.536	35.079	31.503	111.76074	127.5994	112.9	332
14	43.081	42.441	34.711	21.151	177.38555	157.9239	484.2	836.3
15	43.744	44.211	36.784	22.196	161.03830	174.4947	276.9	667.1
17	47.43	42.632	35.924	22.762	137.34864	166.7237	302.2	462.8
18	45.645	43.011	37.099	20.235	192.49248	170.0389	115.3	400.7
19	47.234	44.071	35.906	15.803	111.88793	77.28451	72.1	212.8
20	45.21	43.58	32.594	26.04	161.80963	140.346	181.6	433.6
